# The bacterial quorum sensing peptide iAM373 is a novel inducer of sarcopenia

**DOI:** 10.1002/ctm2.1053

**Published:** 2022-10-13

**Authors:** Anton De Spiegeleer, Evelien Wynendaele, Amélie Descamps, Nathan Debunne, Bart P. Braeckman, Marjan De Mey, Julie Coudenys, Liesbeth Crombez, Frederick Verbeke, Yorick Janssens, Rekin's Janky, Evy Goossens, Caroline Vlaeminck, Dries Duchi, Vanessa Andries, Emilie Dumas, Mirko Petrovic, Tom Van de Wiele, Daniel Knappe, Ralf Hoffmann, Vincent Mouly, Anne Bigot, Lars Vereecke, Filip Van Immerseel, Nele Van Den Noortgate, Bart De Spiegeleer, Dirk Elewaut

**Affiliations:** ^1^ Translational Research in Immunosenescence, Gerontology and Geriatrics (TRIGG) group Ghent University Hospital Ghent Belgium; ^2^ Drug Quality and Registration (DruQuaR) group, Faculty of Pharmaceutical Sciences Ghent University Ghent Belgium; ^3^ VIB Center for Inflammation Research (IRC), Unit for Molecular Immunology and Inflammation Ghent University Ghent Belgium; ^4^ Department of Internal Medicine and Pediatrics, Faculty of Medicine and Health Sciences Ghent University Hospital Ghent Belgium; ^5^ Laboratory for Aging Physiology and Molecular Evolution, Faculty of Sciences Ghent University Ghent Belgium; ^6^ Center for Synthetic Biology, Department of Biotechnology, Faculty of Bioscience Engineering Ghent University Ghent Belgium; ^7^ VIB Nucleomics Core, VIB Leuven Belgium; ^8^ Department of Pathology, Bacteriology and Avian Diseases, Faculty of Veterinary Medicine Ghent University Merelbeke Belgium; ^9^ Host‐Microbiota‐Interaction lab VIB Center for Inflammation Research Ghent Belgium; ^10^ Ghent Gut Inflammation Group (GGIG) Ghent University Ghent Belgium; ^11^ Center for Microbial Ecology and Technology, Faculty of Bioscience Engineering Ghent University Ghent Belgium; ^12^ Center of Biotechnology and Biomedicine, Faculty of Chemistry and Mineralogy University of Leipzig Leipzig Germany; ^13^ Institut de Myologie, Centre de Recherche en Myologie Inserm, Sorbonne Université Paris France

Dear Editor,

Sarcopenia ‐ the accelerated loss of muscle mass, strength and function with ageing – represents an important health challenge with reduced quality of life and increased mortality.^1^ Gut microbiota has been suggested to contribute to this age‐associated muscle wasting but the underlying mechanisms are still unclear.^2^ Here, we uncover the quorum sensing peptide iAM373 as a hitherto unknown contributor to sarcopenia.

In vitro screening has shown that several bacterial quorum sensing molecules can impact C2C12 murine muscle cells, with iAM373 (SIFTLVA), a quorum sensing peptide (QSP) produced by *E. faecalis*, decreasing C2C12 myoblast metabolic activity measured by MTT assays.^3^ Because one of the hallmarks of sarcopenia is a decrease in metabolic activity, we used a stepwise translational approach to evaluate and confirm the relevance of iAM373 in sarcopenia (Figure 1A). Initially, MTT concentration‐response experiments in murine and human myoblasts as well as in myotubes were performed. We found that iAM373 decreased metabolic activity in a concentration‐dependent manner in both murine and human myoblasts and myotubes (Figure 1B and Figure S1). The minimal inhibitory concentrations (IC_10_) for iAM373 to exert these effects were in the pico‐to‐nanomolar range: 61 pM (human) and 282 pM (murine) in myotubes, 35 nM (human) and 10 nM (murine) in myoblasts (cfr. Table S1 for IC_50_ values).

**FIGURE 1:**
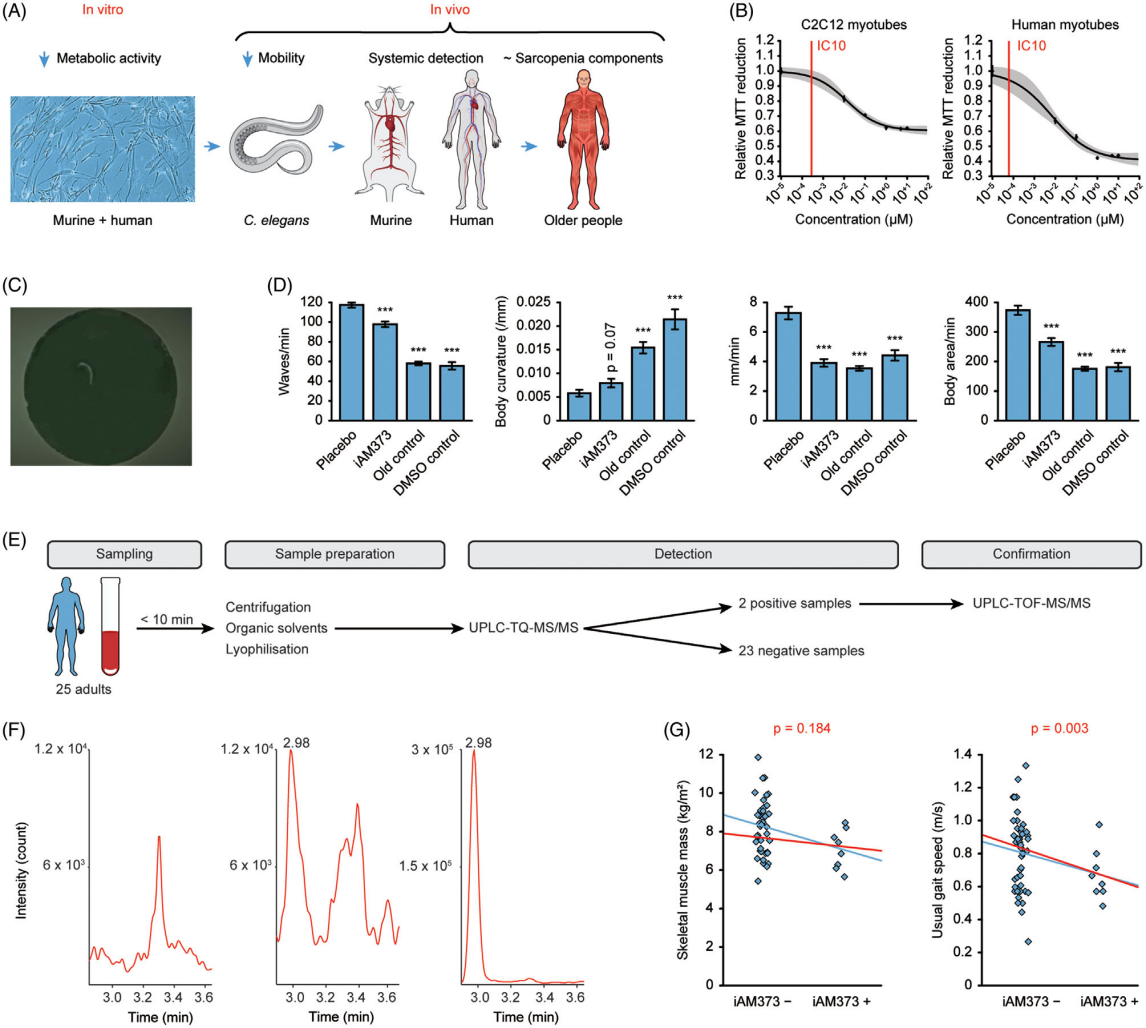
iAM373 is a factor in sarcopenia. (A) Translational approach to investigate the relevance of iAM373. (B) Concentration‐response curve of iAM373 on murine and human myotubes. The x‐axis indicates the different quorum sensing peptide (QSP) concentrations, the y‐axis represents MTT reduction relative to placebo (placebo = 1.0). Squares (+error bars) are means (+SEM) of *n* = 8–10 for each concentration, with the curve representing the best fitting 3‐parameter logistic model around the means. The 95% confidence interval of this curve is shown as grey area. IC_10_ is shown as a red vertical line. (C) Example of a *C. elegans* worm; how it is introduced into automated software. (D) iAM373‐associated changes in mobility variables in wild‐type adults after 3 days incubation with placebo or 1 μM iAM373; sarcopenia controls are older *C. elegans* (12 days with placebo) and *C. elegans* after 3 days incubation with dimethyl sulfoxide (DMSO) 5% (*n* = 216 for placebo; *n* = 146 for iAM373; *n* = 277 for old control; *n* = 155 for DMSO control; from five independent experiments); ***p‐value < .001 (Student's t‐test). (E) Flow chart of human in vivo data acquisition, from a sampling of 25 adult plasma samples to detection and confirmation of iAM373. (F) UPLC‐TQ‐MS/MS precursor (m/z 750.4) → product (*m/z* 449.2) chromatograms of a negative, positive and prespiked (1 nM) human plasma sample for iAM373. (G) Skeletal muscle mass (left) and usual walking speed (right) in the iAM373 positive (*n* = 8) and iAM373 negative (*n* = 43) older cohort. The blue and red lines represent the unadjusted and adjusted regression lines respectively. *p*‐values are indicated for the adjusted regression coefficients

Based on the in vitro results, we examined whether iAM373 is able to induce a sarcopenia phenotype in vivo. We used *C. elegans* to study sarcopenia in vivo as these animals exhibit a sarcopenia phenotype with age that can be measured in an automated manner.^4^, ^5^ Incubation of *C. elegans* worms with iAM373 (1 μM) during 3 days induced a sarcopenia phenotype (Figure 1C,D and Figure S2). The rationale for using this concentration can be found in Text S1. To evaluate the amino acid specificity, we conducted *C. elegans* experiments with a scrambled iAM373 derivative. These experiments confirmed the effects of iAM373 on *C. elegans* mobility as well as demonstrated the primary structure specificity of the observed effects (Figure S3).

Next, we assessed the presence of iAM373 in murine and human plasma. Therefore, we developed a bioanalytical method using reversed‐phase ultra‐high‐performance liquid chromatography coupled to a triple‐quadrupole mass spectrometer (RP‐UHPLC‐TQ‐MS). Positive samples were confirmed using RP‐UHPLC coupled to a time‐of‐flight mass spectrometer (RP‐UHPLC‐TOF‐MS). Method suitability characteristics of this bioanalytical method can be found in Tables S2–4. In a cohort of 25 adults (age, 28 ± 9.3 years; female, 44%), two positive samples were identified (Figure 1E,F). Their estimated concentrations were 16.7 and 77.3 pM. Five of the 25 adults were again sampled on another day – four of them were iAM373 negative on both occasions and one was positive on both occasions (16.7 and 16.0 pM), suggesting low day‐to‐day variation. From a cohort of 15 mice (age, 5 months; male, 100%), one positive sample (1.0 nM) was identified. These findings indicate that iAM373 can be detected in mammalian plasma, and represent the first demonstration of a QSP in human plasma. The presence of iAM373 was in the same order of magnitude (±10% positive) as other QSPs and non‐peptide QSMs in murine and human plasma respectively.^6^, ^7^ Moreover, as we expect that iAM373 can be present in human plasma for years, the anticipated muscle exposures (time x concentration) are similar to the iAM373 exposures causing an effect in the in vitro and *C. elegans* in vivo experiments.

To explore a possible association between the presence of iAM373 and sarcopenia, we recruited fifty‐one residents of an assisted living centre for older people and assessed the presence of iAM373 in plasma as well as skeletal muscle mass, grip strength and walking speed (cfr. Online methods). The main characteristics of the study cohort are presented in Table S5. Of the 51 residents (age, 83 ± 6.9 years; female, 55%), 8(16%) were positive for iAM373; estimated concentrations were between 12 and 453 pM. After multiple adjustments, a clinically and statistically significant association of iAM373 presence with decreased walking speed was demonstrated (‐0.16 m/s; *p* = .003) (Figure 1G and Table S6). In addition, a statistically non‐significant trend of decreased muscle mass with iAM373 presence (‐0.45 kg/m^2^; *p* = .184) was observed.

We further investigated the potential gut origin of iAM373 found in human plasma. While some *E. faecalis* strains isolated from human faeces contain iAM373(Figure S4), a BLAST search did not show the presence of this peptide in the human genome, making the human origin of the plasma iAM373 unlikely. We evaluated the gut permeability to iAM373 in vitro using a CaCo‐2 monolayer permeability assay. At 4 and 37°C, apical‐basal permeability coefficients (P_app_) of 0.87×10^–7^ and 1.4×10^–7^ cm/s respectively were calculated (Figure S5 and Figure 2A). These permeability coefficients are higher than for other bioactive peptides, of which some have been detected in murine plasma in the past.^6^, ^8^, ^9^, ^10^ To explore the potential of iAM373 to cross the gut barrier and reach systemic circulation in vivo, germ‐free mice were inoculated with genetically modified *E. coli* that continuously produce iAM373. When the mice were treated with these bacteria through oral gavage, iAM373 was detected in the blood circulation; mice untreated or treated with unmodified *E. coli* (placebo) did not display iAM373 in their blood (Figure 2B,C).

**FIGURE 2:**
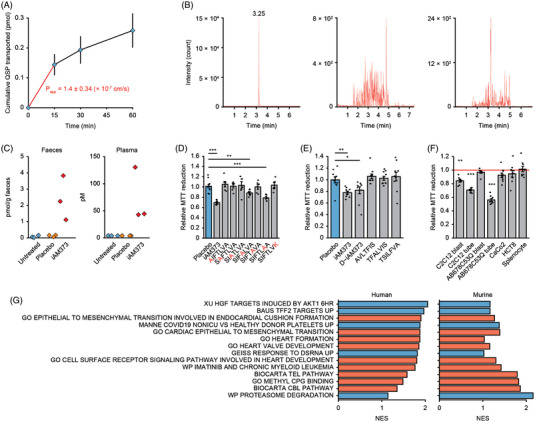
iAM373 crosses the intestinal barrier and selectively impacts muscle cell development and differentiation. (A) iAM373 passes the Caco‐2 monolayer in vitro and accumulates in the acceptor compartment at 37°C (*n* = 6). Data are means ± SEM. (B) Chromatographic profiles of a pre‐spiked plasma sample (left), plasma sample from a germ‐free mouse gavaged with wild‐type *E. coli* (middle) or iAM373‐expressing *E. coli* (right). (C) iAM373 concentrations in faeces and plasma of germ‐free mice untreated (untreated), gavaged with wild‐type *E. coli* (placebo) or gavaged with *E. coli* expressing iAM373 (iAM373). MTT response of C2C12 myotubes after 24 h incubation with (D) placebo, iAM373 or an alanine/lysine derivative at a concentration of 5 μM (*n* = 6 for each condition); (E) placebo, iAM373, D‐configuration derivative, reverse derivative or scrambled derivative (10 μM, *n* = 10 for each condition). (F) MTT response after 24 h incubation of different cell lines with 100 nM of iAM373 (*n* = 9 for each cell line). (D–F) Data are means ± SEM. *p < .05; **p < .01; ***p < .001; two‐tailed Welch's t‐test. (G) RNAsEquation (8 h quorum sensing peptide [QSP] incubation, 1 μM) suggests the effects of iAM373 on muscle development and differentiation. Gene‐set enrichment analysis of iAM373 versus control on C2C12 and human cells using the C2 and C5 curated gene set collection from MSigDB. Gene sets are ordered according to the strength of their enrichment (Normalised Enrichment Score), with blue and red colours indicating positive and negative enrichment respectively; only gene sets with an FDR < 0.25% in both the C2C12 and human cell experiment are represented

To delineate the working mechanisms of iAM373, we evaluated specificity – both at the level of amino acid sequence and cell type ‐ as well as involved pathways. Amino acid specificity experiments using iAM373 derivatives and metabolites identified from ex vivo experiments demonstrated that its activity is sequence‐specific but not chiral‐specific (Figure 2D,E and Text S2). Cell type specificity was explored using two human colonic epithelium cell lines and mouse splenocytes (immune cell mixture). At a concentration of 100 nM, iAM373 did not exert MTT effects on colonic epithelium or immune cells, in contrast to its clear effect on myotubes (Figure 2F). Although more cell lines and concentrations should be evaluated in vitro as well as in vivo to fully assess the degree of cell specificity, these experiments suggest direct iAM373‐muscle interactions causing the observed in vivo metabolic effects. The involved pathways resulting in a decreased muscle metabolism were explored by RNA sequencing of C2C12 and human myotubes after 8‐h treatment with iAM373 or placebo. Gene‐set enrichment analysis (GSEA) revealed enrichment in gene sets involved in striated muscle development and differentiation (Figure 2G). Most of these gene sets were downregulated by iAM373. The proteasome degradation pathway was upregulated in myotubes after QSP incubation (Figure 2G). Merely cytotoxic or cytostatic effects of iAM373 are unlikely, given that viability assays other than the MTT assay did not show a clear cytotoxic or cytostatic effect (Figure S6).

In summary, we unveil the iAM373 quorum sensing peptide as a novel inducer of sarcopenia, opening new perspectives for treating this unmet medical need. Future studies will be needed to thoroughly elucidate the mechanisms of the observed effects as well as elaborate these findings in large prospective human cohorts.

## CONFLICT OF INTEREST

The authors declare that they have no conflict of interest.

## Supporting information



Supporting Information

## References

[1] Cruz‐Jentoft AJ , Bahat G , Bauer J , et al. Sarcopenia: revised European consensus on definition and diagnosis. Age Ageing. 2019;48:16‐31. doi:10.1093/ageing/afy169 30312372 PMC6322506

[2] Liu C , Cheung W‐H , Li J , et al. Understanding the gut microbiota and sarcopenia: a systematic review. J Cachexia Sarcopenia Muscle. 2021;12:1393‐1407. doi:10.1002/jcsm.12784 34523250 PMC8718038

[3] De Spiegeleer A , Elewaut D , Van Den Noortgate N , et al. Quorum sensing molecules as a novel microbial factor impacting muscle cells. Biochim Et Biophys Acta‐Mol Basis Dis. 2020;1866:165646. doi:10.1016/j.bbadis.2019.165646 31870715

[4] Christian CJ , Benian GM . Animal models of sarcopenia. Aging Cell. 2020;19. doi:10.1111/acel.13223 PMC7576270 32857472

[5] Restif C , Ibáñez‐Ventoso C , Vora MM , Guo S , Metaxas D , Driscoll M . CeleST: computer vision software for quantitative analysis of C. elegans swim behavior reveals novel features of locomotion. Plos Comput Biol. 2014;10:e1003702. doi:10.1371/journal.pcbi.1003702 25033081 PMC4102393

[6] Wynendaele E , Debunne N , Janssens Y , et al. The quorum sensing peptide EntF* promotes colorectal cancer metastasis in mice: a new factor in the host‐microbiome interaction. BMC Biol. 2022;20:151‐151. doi:10.1186/s12915-022-01317-z 35761265 PMC9238271

[7] Verbeke F , De Craemer S , Debunne N , et al. Peptides as quorum sensing molecules: measurement techniques and obtained levels in vitro and in vivo. Front Neurosc. 2017;11:1‐18. doi:10.3389/fnins.2017.00183 PMC5388746 28446863

[8] Janssens Y , Debunne N , De Spiegeleer A , et al. PapRIV, a BV‐2 microglial cell activating quorum sensing peptide. Sci Rep. 2021;11:10723. doi:10.1038/s41598-021-90030-y PMC8140105 34021199

[9] De Spiegeleer B , Verbeke F , D'Hondt M , et al. The quorum sensing peptides PhrG, CSP and EDF promote angiogenesis and invasion of breast cancer cells in vitro. Plos One. 2015;10:e0119471. doi:10.1371/journal.pone.0119471 25780927 PMC4363635

[10] Lin QL , Xu Q , Bai J , Wu W , Hong H , Wu J . Transport of soybean protein‐derived antihypertensive peptide LSW across Caco‐2 monolayers. J Functional Foods. 2017;39:96‐102. doi:10.1016/j.jff.2017.10.011

