# Supplementary material for: The bacterial quorum sensing peptide iAM373 is a novel inducer of sarcopenia
Source: Clin Transl Med. 2022 Oct 13;12(10):e1053. doi: 10.1002/ctm2.1053 (PMC9561422; doi:10.1002/ctm2.1053)
Supplement: Supplementary file 1 — Supporting Information [file CTM2-12-e1053-s001.docx]

**Supplementary Information for**

**The bacterial Quorum Sensing Peptide iAM373 is a novel inducer of sarcopenia.**

*Anton De Spiegeleer^1,2,3,4^, Evelien Wynendaele^1,2^, Amélie Descamps^1,2^, Nathan Debunne^2^, Bart P. Braeckman^5^, Marjan De Mey^6^, Julie Coudenys^1,3,5^, Liesbeth Crombez^1,4^, Frederick Verbeke^2^, Yorick Janssens^2^, Rekin’s Janky^7^, Evy Goossens^8^, Caroline Vlaeminck^5^, Dries Duchi^6^, Vanessa Andries^3,4,9,10^, Emilie Dumas^3,4^, Mirko Petrovic^4^, Tom Van de Wiele^11^, Ralf Hoffmann^12^, Vincent Mouly^13^, Anne Bigot^13^, Lars Vereecke^3,4,9,10^, Filip Van Immerseel^9^, Nele Van Den Noortgate^1,4^, Bart De Spiegeleer^2*^ and Dirk Elewaut^1,3,4*^*

^1^ Translational Research in Immunosenescence, Gerontology and Geriatrics (TRIGG) group, Ghent University Hospital, Ghent, Belgium.

^2^ Drug Quality and Registration (DruQuaR) group, Faculty of Pharmaceutical Sciences, Ghent University, Ghent, Belgium.

^3^ VIB Inflammation Research Center, Unit for Molecular Immunology and Inflammation, Ghent University, Ghent, Belgium.

^4^ Department of Internal Medicine and Pediatrics, Faculty of Medicine and Health Sciences, Ghent University Hospital, Ghent, Belgium.

^5^ Laboratory for Aging Physiology & Molecular Evolution, Faculty of Sciences, Ghent University, Ghent, Belgium.

^6^ Center for Synthetic Biology, Department of Biotechnology, Faculty of Bioscience Engineering, Ghent University, Ghent, Belgium.

^7^ VIB Nucleomics Core, VIB, Leuven, Belgium.

^8^ Department of Pathology, Bacterioly & Avian Diseases, Faculty of Veterinary Medicine, Ghent University, Merelbeke, Belgium.

^9^ Host-Microbiota-Interaction lab, VIB Center for Inflammation Research, Ghent, Belgium

^10^ Ghent Gut Inflammation Group (GGIG), Ghent University, Ghent, Belgium

^11^ Center for Microbial Ecology and Technology, Faculty of Bioscience Engineering, Ghent University, Ghent, Belgium.

^12^ Center of Biotechnology and Biomedicine, Faculty of Chemistry and Mineralogy, University of Leipzig, Leipzig, Germany.

^13^ Institut de Myologie, Sorbonne Université, Paris, France.

* shared corresponding authorship

e-mail: bart.despiegeleer@ugent.be; dirk.elewaut@ugent.be

**Content:**

Supplementary Figures 1-6 Pages 2-7

Supplementary Tables 1-6 Pages 8-11

Supplementary Text 1 Pages 12-16

**Supplementary Figure 1**


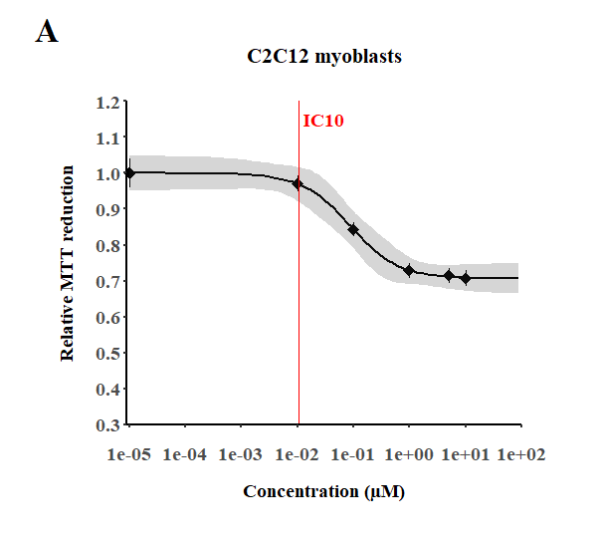

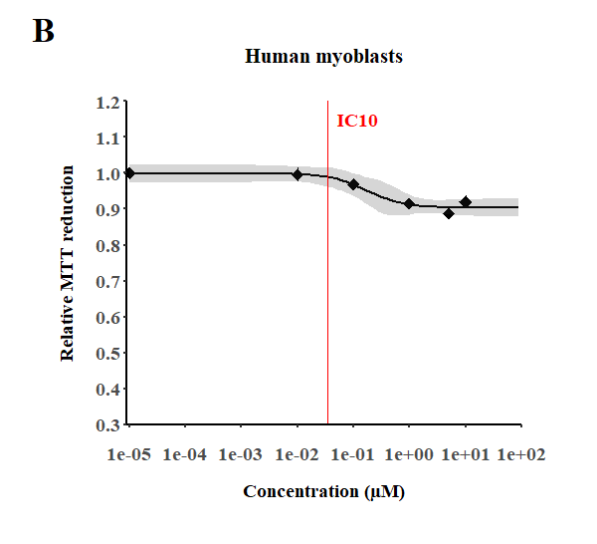


Supplementary Figure 1. Concentration response curve of iAM373 on murine and human myoblasts. A) C2C12 myoblasts; B) human myoblasts. The x-axis indicates the different QSP concentrations, while the y-axis represents MTT reduction relative to placebo (placebo = 1.0). Squares (+ error bars) are means (+ SEM) of n=8-10 for each concentration, with the curve representing the best fitting 3-parameter logistic model around the means. The 95% confidence interval of this curve is shown as grey area. IC10 is shown as a red vertical line.

**Supplementary Figure 2**


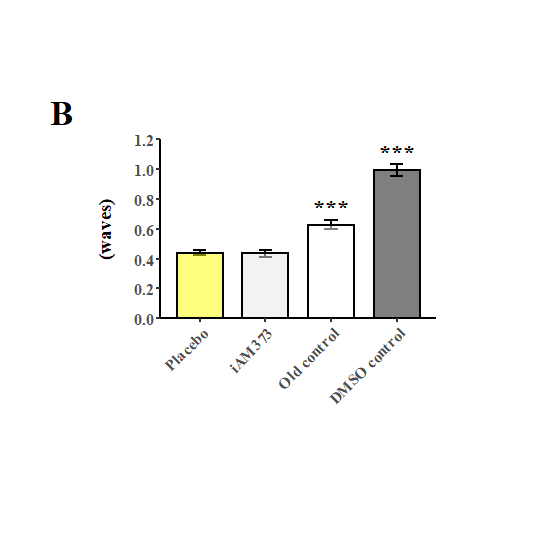

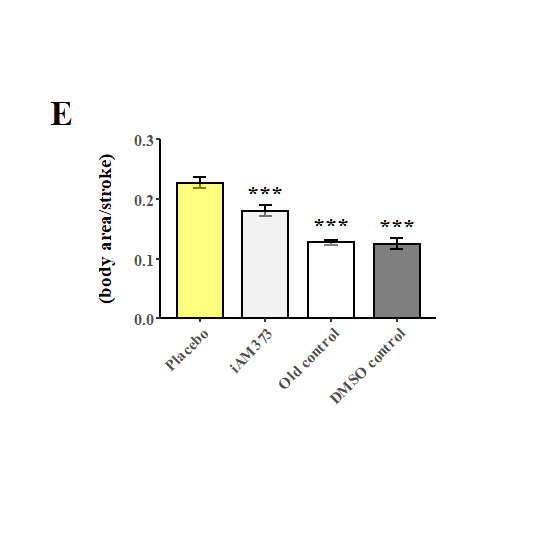


Supplementary Figure 2: iAM373 induces a sarcopenic muscle phenotype in *C. elegans*, without a clear effect on survival. a, b) iAM373-associated changes in swimming parameters in wild-type adults after 3 days incubation with placebo or 1 µM iAM373; positive controls are aged *C. elegans* (12 days with placebo) and *C. elegans* after 3 days incubation with DMSO 5% (n = 216 for placebo; n = 146 for iAM373; n = 277 for old control; n = 155 for DMSO control; from 5 independent experiments). a) Body wave number; b) Brush stroke. c, d) Survival experiments of adult worms treated once with placebo, 1 µM iAM373 or DMSO 5% (n = 363 for placebo; n = 341 for iAM373; n = 368 for DMSO control; from 5 independent experiments). c) Kaplan–Meier survival analysis. d) Mean lifespan. Data presented are means ± SEM. * P < 0.05; ** P < 0.01 and *** P < 0.001; Welch’s 2-sided t-test with placebo as reference.

**D**

**A**


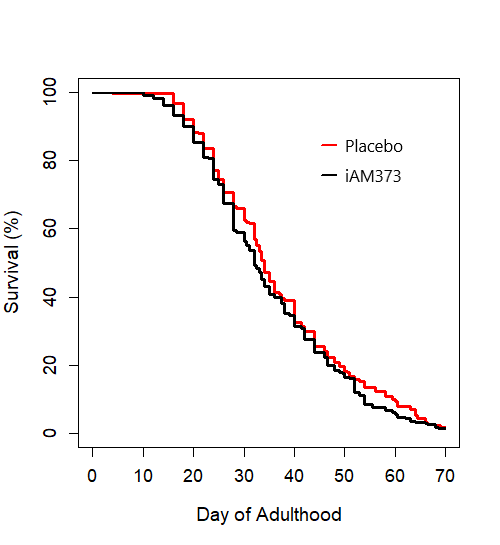


**C**


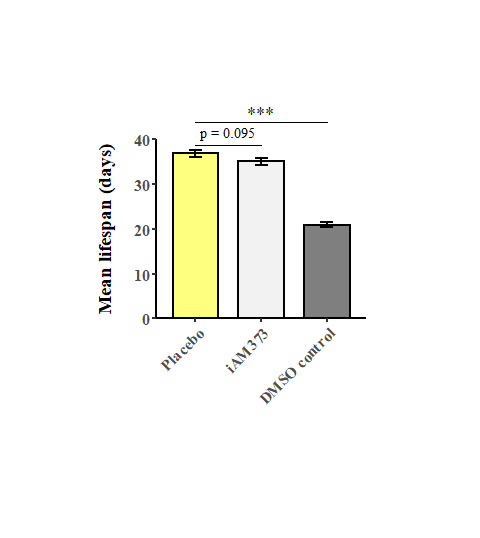


**B**

**Supplementary Figure 3**


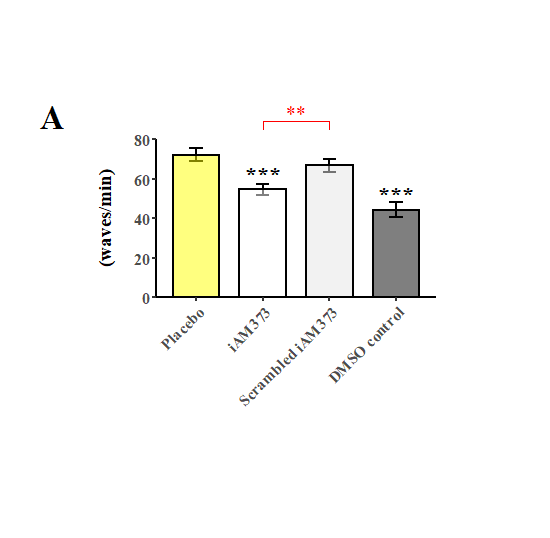

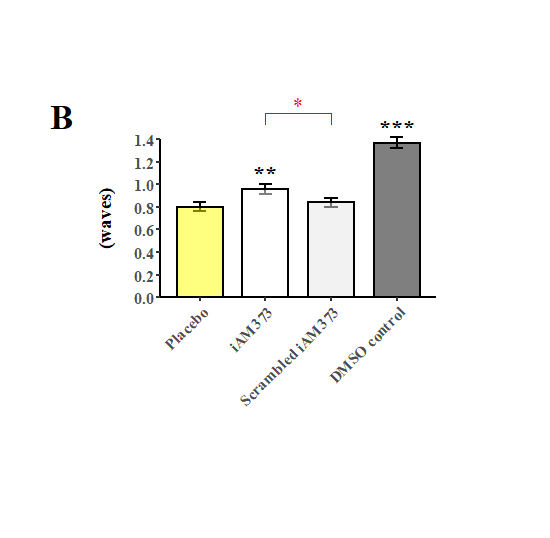

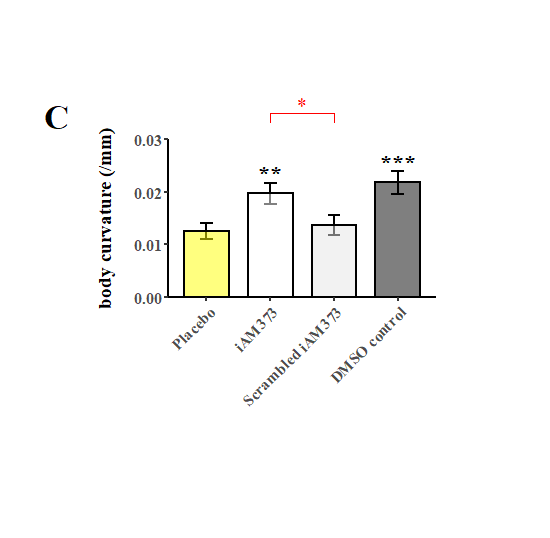

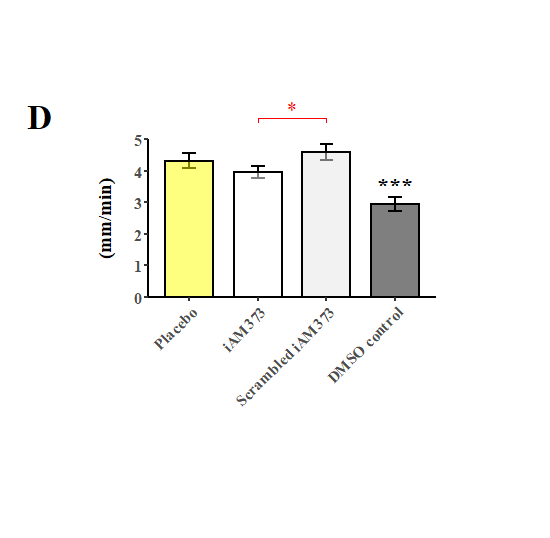

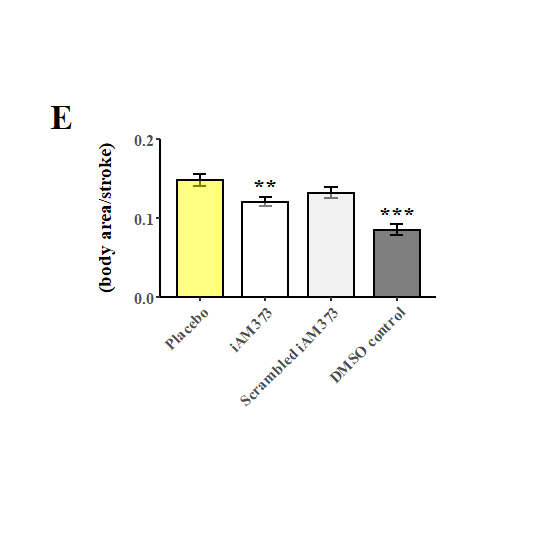

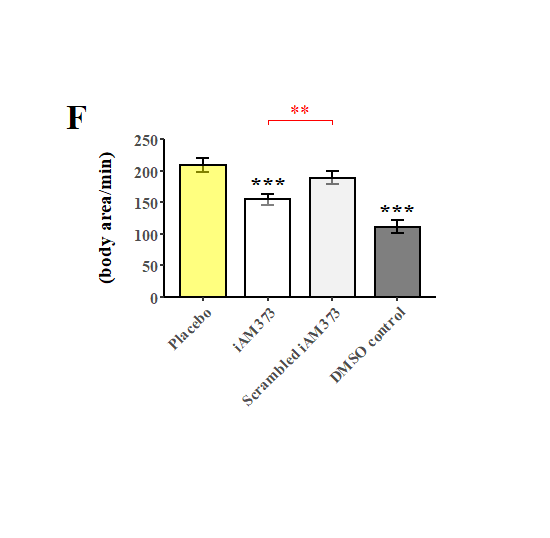


Supplementary Figure 3. iAM373 induces an aging muscle phenotype in C. elegans. (a-f) iAM373-associated changes in swimming parameters in wild-type adults after 3 days incubation with placebo, 1 µM iAM373 or 1 µM scrambled iAM373; positive controls are aged *C. elegans* after 3 days incubation with DMSO 5%. a) Wave initiation rate. b) Body wave number. c) Asymmetry. d) Travel speed. e) Brush stroke. f) Activity index. Data presented are means ± SEM. * P < 0.05; ** P < 0.01 and *** P < 0.001; Welch’s 2-sided t-test with black asterisks using Placebo as a reference and red asterisks using iAM373 as a reference (n = 149 for placebo; n = 161 for iAM373; n = 151 for scrambled iAM373; n = 140 for DMSO control; from 3 independent experiments).

**Supplementary
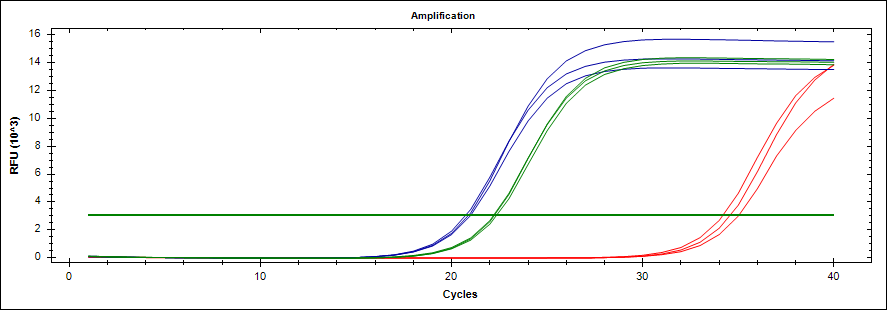
Figure 4**

Supplementary Figure 4. iAM373 DNA qPCR amplification curves of iAM373 containing E. faecalis strains 28157_4#179 (green) and ATCC 43062 (blue), as well as the iAM373 negative E. faecalis strain immac69 (red).


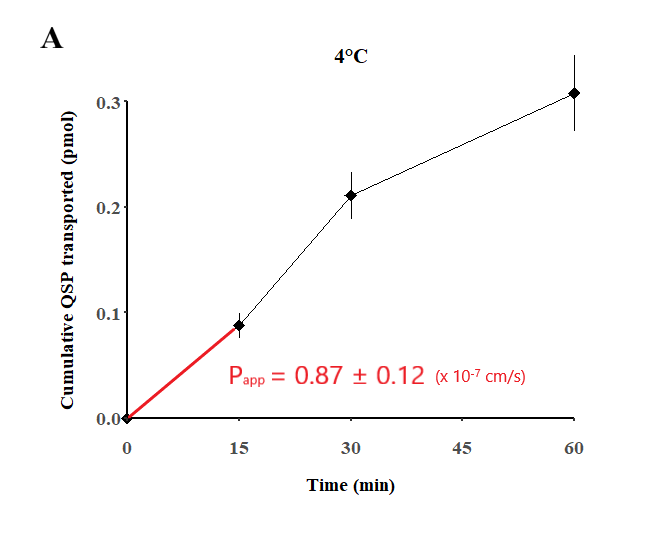
**Supplementary Figure 5**

Supplementary Figure 5. The iAM373 peptide passes the Caco-2 monolayer in vitro and accumulates in the acceptor compartment at 4 °C (n = 6). Data are means ± SEM.

**Supplementary Figure 6**


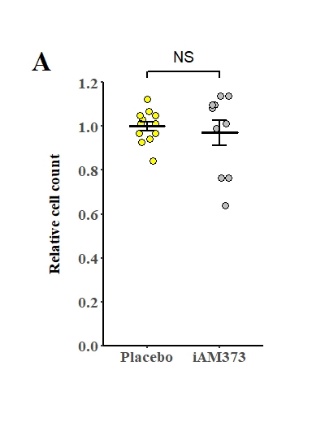

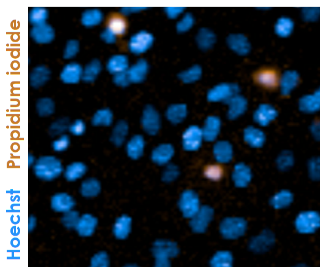

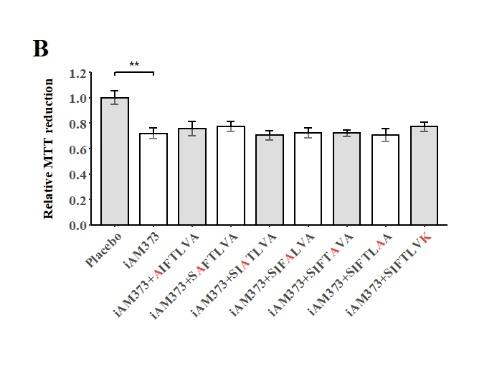

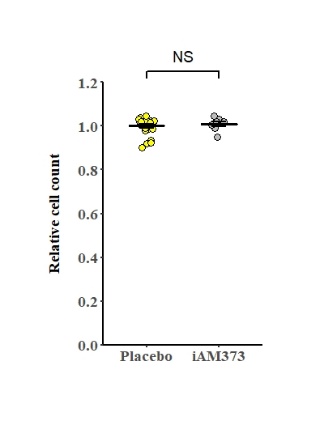

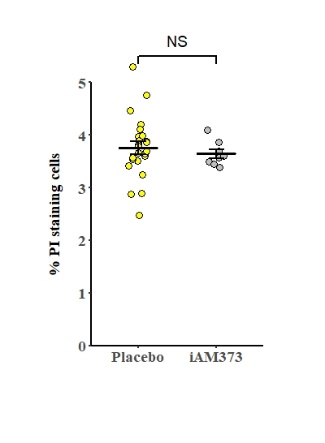

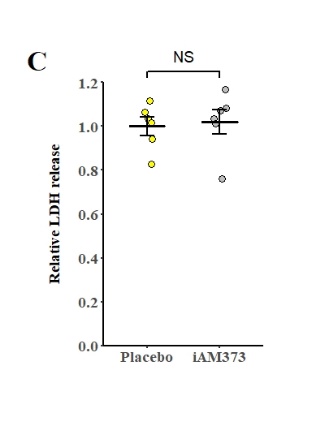

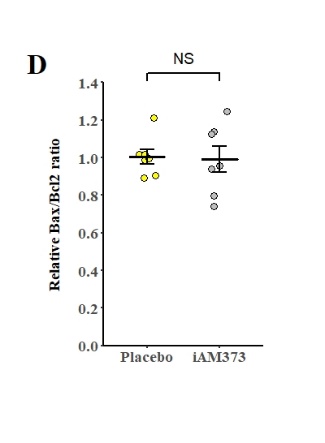

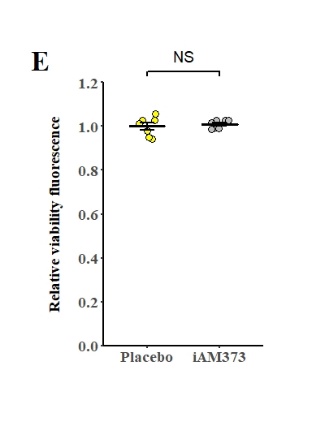

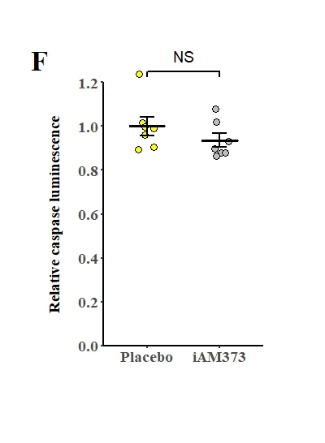


Supplementary Figure 6. The MTT effects of iAM373 are not merely cytotoxic or cytostatic. Different viability assays were conducted after C2C12 myoblast incubation with iAM373 at 1 µM. a) Relative cell count (manual) using trypan blue and light microscopy. b) A representative image, the relative cell count (automated) and % of death cells using Hoechst/PI staining. c) Relative LDH release using colorimetry. d) mRNA expression of Bax relative to Bcl2 as marker for apoptosis. e) Relative viability fluorescence using Apotox assay. f) Relative caspase luminescence using Apotox assay.

**Supplementary Table 1**

**Supplementary Table 1:** IC_50_ values with 95% confidence intervals (CI) for iAM373 dose-response curves on muscle cells.

| **Cell line** | **IC_50_** | **CI for IC_50_** |
| --- | --- | --- |
| Human myotubes | 5.4 nM | 22 pM to 12 nM |
| Murine myotubes | 13 nM | 4.8 nM to 25 nM |
| Human myoblasts | 180 nM | 41 nM to 831 nM |
| Murine myoblasts | 61 nM | 20 nM to 162 nM |

**Supplementary Tables 2-4**

**Supplementary Table 2:** Average recovery, matrix effect and process efficiency of iAM373 in human plasma determined on Waters Xevo™ TQ-S triple quadrupole mass spectrometer with electrospray ionization (operated in positive ionization mode).

| **Parameter** | **Matrix 1** | **Matrix 2** | **Matrix 3** | **Overall** | **SD** | **RSD** |
| --- | --- | --- | --- | --- | --- | --- |
| Process yield | **70.5%** | | | | | |
| Recovery | 67.7% | 64.7% | 58.7% | **63.7%** | 4.6% | 7.2% |
| Matrix Effect | 85.3% | 65.1% | 60.3% | **70.2%** | 13.3% | 18.9% |
| Process efficiency | 57.7% | 42.1% | 35.4% | **45.1%** | 11.5% | 25.4% |

The limit of detection of the whole analytical process was determined based on S/N=3 ratio of the 1 nM and 10 pM prespiked plasma samples of the quantifier (3 different matrices/concentration).

**Supplementary Table 3**: Determined Limit of detection (LoD) of iAM373.

| **S/N ǀ LoD at 1 nM** | | | **S/N ǀ LoD at 10 pM** | | | **LoD @ 10pM (SD)** |
| --- | --- | --- | --- | --- | --- | --- |
| **M1** | **M2** | **M3** | **M4** | **M5** | **M6** | 6.8 (0.5) pM |
| 288 **ǀ** 10.4 pM | 360 **ǀ** 8.3pM | 252 **ǀ** 12.0 pM | 4.8 **ǀ** 6.3 pM | 4.4 **ǀ** 6.8 pM | 4.1 **ǀ** 7.3 pM |  |

The limit of identification of the whole analytical process was determined based on S/N=3 ratio of 1 nM and 10 pM prespiked plasma samples of the qualifier (3 different matrices/concentration).

**Supplementary Table 4:** Determined Limit of identification (LoI) of iAM373.

| **S/N ǀ LoI at 1 nM** | | | **S/N ǀ LoI at 10 pM** | | | **LoI @ 10pM (SD)** |
| --- | --- | --- | --- | --- | --- | --- |
| **M1** | **M2** | **M3** | **M4** | **M5** | **M6** | **8.1 (2) pM** |
| **290 ǀ 10.3 pM** | **274 ǀ 10.9 pM** | **212 ǀ 14.2 pM** | 4.6 **ǀ** 6.5 pM | 4.0 **ǀ** 7.5 pM | 2.9**ǀ**10.3 pM |  |

The reporting threshold was defined as the highest determined LoI of the 10 pM prespiked samples.

**Supplementary Table 5**

**Supplementary Table 5**. Characteristics of the study cohort. Categorical variables are shown as numbers (% of column N), while continuous variables are means (standard deviation or SD).

| **Sample characteristics** | **Total**  **(N = 51)** | **iAM373 positive***  **(N = 8)** | **iAM373 negative**  **(N = 43)** |
| --- | --- | --- | --- |
| Age in years, mean (SD) | 83.0 (6.9) | 84.8 (6.7) | 82.7 (6.9) |
| Male, n (%) | 23 (45.1) | 1 (12.5) | 22 (51.2) |
| BMI in kg/m², mean (SD) | 29.0 (5.6) | 28.3 (2.6) | 29.1 (6.1) |
| MMSE, mean (SD) | 26.1 (3.8) | 27.1 (3.5) | 25.9 (3.9) |
| PASE-score, mean (SD) | 52.4 (33.6) | 60.4 (30.4) | 51.0 (34.3) |
| SM in kg, mean (SD) | 21.0 (5.3) | 17.7 (3.4) | 21.7 (5.4) |
| SM height corrected in kg/m², mean (SD) | 8.0 (1.4) | 7.1 (1.0) | 8.2 (1.4) |
| Grip strength in kg, mean (SD) | 24.4 (7.9) | 22.4 (6.4) | 24.8 (8.1) |
| Usual walking speed in m/s, mean (SD) | 0.79 (0.23) | 0.67 (0.16) | 0.81 (0.23) |

* ≥ reporting threshold of 10 pM

**Supplementary Table 6**

**Supplementary Table 6.** Summary of coefficients (Coeff) for muscle mass, grip strength and usual walking speed (iAM373 positive vs. negative) using propensity score overlap weighted linear regression (n=51).*

| Sarcopenia component | Coeff (95% CI)  iAM373 positive vs. negative | P-value |
| --- | --- | --- |
| Muscle mass (kg/m²) | -0.45 (-1.10; 0.20) | 0.184 |
| Grip strength (kg) | 1.85 (-1.70; 5.40) | 0.312 |
| Usual walking speed (m/s) | -0.16 (-0.26; -0.06) | **0.003** |

* Minimal clinical important differences in absolute values for height-adjusted muscle mass (kg/m²), grip strength (kg) and usual walking speed (m/s) are estimated 0.5 kg/m², 5.0 kg and 0.10 m/s ^1-3^.

1 Pichard, C. *et al.* Nutritional assessment: lean body mass depletion at hospital admission is associated with an increased length of stay. *American Journal of Clinical Nutrition* **79**, 613-618 (2004).

2 Bohannon, R. W. Minimal clinically important difference for grip strength: a systematic review. *Journal of physical therapy science* **31**, 75-78, doi:10.1589/jpts.31.75 (2019).

3 Bohannon, R. W. & Glenney, S. S. Minimal clinically important difference for change in comfortable gait speed of adults with pathology: a systematic review. *Journal of Evaluation in Clinical Practice* **20**, 295-300, doi:10.1111/jep.12158 (2014).

**Supplementary Text 1**

For the functional in-vivo experiments, we chose an iAM373 concentration where maximal effect can be expected based on the in-vitro experiments, i.e. a concentration around the IC90 (IC90 of human myoblasts is 0.93 µM). Given this is a proof-of-concept study aiming to explore if iAM373 can clearly affect functional sarcopenia outcomes (without measuring small effects), this seems appropriate.

Moreover, a starting concentration of 1 µM in C. elegans medium during a short time period such as 3 days will result in an exposure similar to lower concentrations during longer time periods. This seems reasonable because we expect the plasma iAM373 concentration in human plasma to be present for a long time to induce gradual muscle wasting in sarcopenia.

**Supplementary Text 2: ex-vivo metabolization experiments**

*Ex vivo* murine metabolization studies were carried out to identify and assess the biological activity of the main metabolites, as well as to assess the stability of iAM373 in different matrices. iAM373 was found to be most stable in colon, followed by muscle, liver, serum, faeces and kidney homogenate respectively (**Supplementary Table T2.1**). Four main metabolites were identified; however, they did not reduce metabolic activity (MTT assay) in C2C12 myotubes (**Supplementary Figure T2.1**).

Supplementary Table T2.1. Half-life and main metabolites of iAM373 in different murine matrices ex vivo.

| **Tissue** | **Calculated half-life (min) [95% C.I.]** | **Main metabolite**  **at approx. T_1/2_** |
| --- | --- | --- |
| Liver | 163 [146, 183] | FTLVA (180 min) |
| Colon | 215 [171, 292] | SIFTL (180 min) |
| Muscle | 199 [93.5, -] | SIFTL (180 min) |
| Kidney | 31.6 [21.1, 62.7] | SIFT (30 min)  SIFTL (30 min) |
| Faeces | 51.4 [33.1, 114] | SIF (60 min) |
| Serum | 131 [87.2, 260] | -^(1)^ |

(1) No metabolites could be identified due to co-eluting blank peaks

**
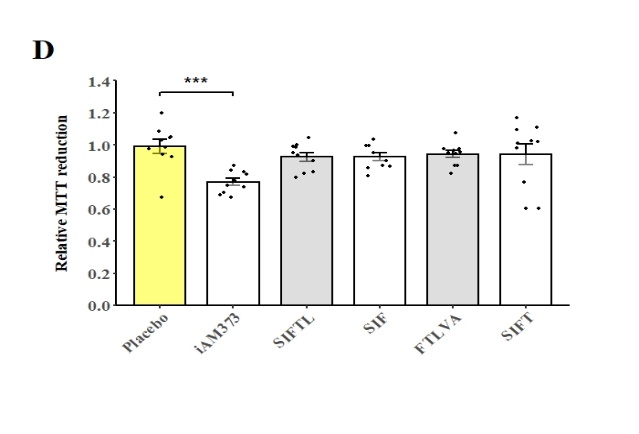
**

**Supplementary Figure T2.1**. MTT response of C2C12 myotubes after 24h incubation with placebo, iAM373 or one of its main metabolites (10 µM, n=10 for each condition). Data are means ± SEM. *P < 0.05; **P < 0.01; ***P < 0.001; two-tailed Welch’s t-test.

**Protocol**

*Homogenate preparation*

Preparation of Krebs-Henseleit buffer (pH 7.4) (KH-buffer)

The powdered medium is dissolved in 900 mL water while stirring. To this solution, 0.3790 g CaCl_2_×2H_2_O and 2.098 g NaHCO_3_ are subsequently added while stirring. NaOH or HCl was used to adjust to pH 7.4. This solution is then further diluted to 1000 mL using ultrapure water.

*Preparation of serum*

The blood was collected in a 1.5 mL Eppendorf tube after decapitating the mouse. The blood of the 4 mice was pooled and centrifuged for 10 min at 1000 g at 5 °C. The supernatant (serum) was transferred to a 500 µL Eppendorf and was immediately frozen and stored at -35 °C.

*Preparation of liver homogenate*

The liver (5.0070 g) was collected from four C57BL/6 mice after cervical dislocation. After cleaning and rinsing the organ using ice-cold KH buffer, the liver was cut in little pieces and transferred into a 50 mL tube to which 36 mL ice-cold KH buffer was added. The liver was then homogenized with mixer. After the larger particles were allowed to settle for about 30 minutes at 5 °C, approximately 25 mL of the middle layer was transferred into a 50 mL Falcon tube using a plastic pipette. After shaking the homogenate, the homogenate was dispensed into ten 2 mL Eppendorf tubes, and stored at -35 °C until use. The protein content of the homogenate was determined to be 18.24 mg/mL using the Pierce Modified Lowry Protein Assay method. Just before use, the homogenate was diluted to a protein concentration of 0.6 mg/mL (*i.e.* 300 µg / 500 µl) by the addition of 29.4 mL of KH buffer to 1000 µl of the homogenate.

*Preparation of kidney homogenate*

The kidneys (1.4143 g) were collected from four C57BL/6 mice after cervical dislocation. After cleaning and rinsing the organs using ice-cold KH buffer, the kidneys were cut in little pieces and transferred into a 50 mL tube to which 24 mL ice-cold KH buffer was added. The kidneys were then homogenized with mixer. After the larger particles were allowed to settle for about 30 minutes at 5 °C, approximately 10 mL of the middle layer was transferred into a 50 mL Falcon tube using a plastic pipette. After shaking the homogenate, the homogenate was dispensed into five 2 mL Eppendorf tubes, and stored at -35 °C until use. The protein content of the homogenate was determined to be 5.92 mg/mL using the Pierce Modified Lowry Protein Assay method. Just before use, the homogenate was diluted to a protein concentration of 0.6 mg/mL (*i.e.* 300 µg / 500 µl) by the addition of 8.87 mL of KH buffer to 1000 µl of the homogenate.

*Preparation of colon homogenate*

The colons (incl. caecum) (1.4174 g) were collected from four C57BL/6 mice after cervical dislocation. After cleaning and rinsing the organs using ice-cold KH buffer, the colons were cut in little pieces and transferred into a 15 mL tube to which 5 mL ice-cold KH buffer was added. The colons were then homogenized with mixer. After the larger particles were allowed to settle for about 30 minutes at 5 °C, approximately 2 mL of the middle layer was dispensed into a 2 mL Eppendorf tube, and stored at -35 °C until use. The protein content of the homogenate was determined to be 2.35 mg/mL using the Pierce Modified Lowry Protein Assay method. Just before use, the homogenate was diluted to a protein concentration of 0.6 mg/mL (*i.e.* 300 µg / 500 µl) by the addition of 2.92 mL of KH buffer to 1000 µl of the homogenate.

*Preparation of faeces homogenate*

Fresh faeces (0.1810 g) was collected from four C57BL/6 mice. The faeces was then cut in little pieces and transferred into a 15 mL tube to which 5 mL ice-cold KH buffer was added. The faeces was then homogenized with mixer. After the larger particles were allowed to settle for about 30 minutes at 5 °C, approximately 2 mL of the middle layer was dispensed into a 2 mL Eppendorf tube, and stored at -35 °C until use. The protein content of the homogenate was determined to be 1.48 mg/mL using the Pierce Modified Lowry Protein Assay method. Just before use, the homogenate was diluted to a protein concentration of 0.6 mg/mL (*i.e.* 300 µg / 500 µl) by the addition of 1.47 mL of KH buffer to 1000 µl of the homogenate.

*Preparation of muscle homogenate*

Muscle (0.6265 g) was collected from four C57BL/6 mice after cervical dislocation. After cleaning and rinsing the organs using ice-cold KH buffer, the muscle was cut in little pieces and transferred into a 15 mL tube to which 5 mL ice-cold KH buffer was added. The muscles were then homogenized with mixer. After the larger particles were allowed to settle for about 30 minutes at 5 °C, approximately 2 mL of the middle layer was dispensed into a 2 mL Eppendorf tube, and stored at -35 °C until use. The protein content of the homogenate was determined to be 6.15 mg/mL using the Pierce Modified Lowry Protein Assay method. Just before use, the homogenate was diluted to a protein concentration of 0.6 mg/mL (*i.e.* 300 µg / 500 µl) by the addition of 9.24 mL of KH buffer to 1000 µl of the homogenate.

*UHPLC instrumental method*

Following conditions are used during the analyses:

Column: Acquity UHPLC® BEH C18 (2.1 x 100 mm; 1.7 µm) + guard column (column U2)

Sample compartment: 10 ± 3 °C

Column temperature: 60 ± 5 °C

Injection volume: 10.0 µL

Mobile phase A: water/ACN 95/5 (V/V) + 0.1 % FA (m/V)

Mobile phase B: water/ACN 5/95 (V/V) + 0.1 % FA (m/V)

Needle wash: water/ACN/DMSO (45/45/10) (V/V/V) +0.1 % FA (m/V), post-injection during 6 seconds

Purge solvent: MeOH

Seal wash: MeOH/water (10/90) (V/V)

**Supplementary Table T2.2:** Mobile phase gradient of the chromatographic system.

| **Time (min)** | **Flow rate (mL/min)** | **Solvent composition** | |
| --- | --- | --- | --- |
|  |  | **MPA (%)** | **MPB (%)** |
| Initial | 0.500 | 100.0 | 0.0 |
| 1.00 |  | 100.0 | 0.0 |
| 10.00 |  | 40.0 | 60.0 |
| 10.50 |  | 14.2 | 85.8 |
| 11.50 |  | 14.2 | 85.8 |
| 11.51 |  | 100.0 | 0.0 |
| 15.00 |  | 100.0 | 0.0 |
